# Cyclic guanosine monophosphate improves salt tolerance in *Solanum lycopersicum*

**DOI:** 10.1007/s10265-023-01487-z

**Published:** 2023-08-23

**Authors:** Gulnaz Bibi, Iqra Shafique, Sartaj Ali, Raza Ahmad, Mohammad Maroof Shah, Tatheer Alam Naqvi, Iftikhar Zeb, Frans J. M. Maathuis, Jamshaid Hussain

**Affiliations:** 1 Department of Biotechnology, COMSATS University Islamabad, Abbottabad Campus, University Road, Tobe Camp, Abbottabad, 22060 Pakistan; 2 Biology Department, University of York, York, YO10 5DD UK

**Keywords:** Cation fluxes, Cyclic nucleotide, Electrolyte leakage, Salt stress

## Abstract

The cyclic nucleotide cyclic guanosine monophosphate (cGMP) is a powerful cell signaling molecule involved in biotic and abiotic stress perception and signal transduction. In the model plant *Arabidopsis thaliana*, salt and osmotic stress rapidly induce increase in cGMP which plays role by modulating the activity of monovalent cation transporters, possibly by direct binding to these proteins and by altering the expression of many abiotic stress responsive genes. In a recent study, a membrane permeable analogue of cGMP (8-bromo-cGMP) was found to have a promotive effect on soluble sugar, flavonoids and lignin content, and membrane integrity in *Solanum lycopersicum* seedlings under salt stress. However, it remains to be elucidated how salt stress affects the endogenous cGMP level in *S. lycopersicum* and if Br-cGMP-induced improvement in salt tolerance in *S. lycopersicum* involves altered cation fluxes. The current study was conducted to answer these questions. A rapid increase (within 30 s) in endogenous cGMP level was determined in *S. lycopersicum* roots after treatment with 100 mM NaCl. Addition of membrane permeable Br-cGMP in growth medium remarkably ameliorated the inhibitory effects of NaCl on seedlings’ growth parameters, chlorophyll content and net photosynthesis rate. In salt stressed plants, Br-cGMP significantly decreased Na^+^ content by reducing its influx and increasing efflux while it improved plants K^+^ content by reducing its efflux and enhancing influx. Furthermore, supplementation with Br-cGMP improved plant’s proline content and total antioxidant capacity, resulting in markedly decreased electrolyte leakage under salt stress. Br-cGMP increased the expression of Na^+^/H^+^ antiporter genes in roots and shoots of *S. lycopersicum* growing under salt stress, potentially enhancing plant’s ability to sequester Na^+^ into the vacuole. The findings of this study provide insights into the mechanism of cGMP-induced salt stress tolerance in *S. lycopersicum*.

## Introduction

Soil salinity is one of the major abiotic stressors that reduces the global agricultural output. Salinity has affected more than 800 million hectares of agricultural land worldwide (Liu and Wang 2021). Saline conditions affect plant’s architecture, metabolism, biochemistry, gene transcription and many other essential processes (Hassan et al. 2021). Since most crops, including tomato, are glycophytes, their growth and development are negatively affected by salt stress, ultimately causing extensive yield losses. Salt stress causes damage to plants mainly through three different ways. First of all, plants are subjected to osmotic stress which creates water deficiency. Secondly, toxic ions accumulate in plants that damage sensitive cellular macromolecules and disrupt cellular metabolism. Third aspect includes excessive production of reactive oxygen species leading to oxidative stress which affects membrane permeability, lipid peroxidation and electrolyte leakage (Souza et al. 2019).

Being sessile, plants must devise strategies for adaptation to saline environments. These include osmotic adjustment by accumulation of compatible solutes, establishment of ion homeostasis, and scavenging reactive oxygen species (ROS). Osmotic adjustment is carried out by synthesizing compatible solutes like proline and glycine betaine to maintain cell turgor and volume (Van Zelm et al. 2020). Through osmotic adjustment plants prevent dehydration stress by reducing the cytosolic osmotic potential (Yang and Guo 2018; Jogawat 2019). In plants, most of the salt tolerance mechanisms are associated with transport and compartmentalization of sodium ions. Na^+^ /H^+^ antiporter (*NHX*) is involved in the transport of Na^+^ ions from cytoplasm to vacuole or outside of the cell. To achieve this, it utilizes the H^+^ electrochemical gradient formed by two proton pumps, i.e., H^+^ -ATPase and H^+^ -PPase thereby preventing the cell from the toxic effects of sodium ions (Apse et al. 1999). Plant’s potassium (K^+^) content is one of the important determinants of salt stress tolerance (Shabala and Cuin 2008). A strong positive correlation exists between the K^+^ retention ability under salt stress and overall plants salt tolerance (Cuin et al. 2008). Different K^+^ transporters and channels mediate high/low-affinity K^+^ uptake in plants. The high affinity K^+^ transporter 5 (*HAK5*), is localized in the root epidermis and is associated with K^+^ accumulation in Arabidopsis (Harada and Leigh 2006). Antioxidant defense system comprising of enzymatic (SOD, POD, CAT) and non-enzymatic antioxidants (phenolic acids, flavonoids, carotenoids, non-protein amino acids) (Hasanuzzaman et al. 2019) functions to scavenge ROS (Czarnocka and Karpiński 2018). Under salt stress photosynthetic capacity is reduced, cell division and expansion are inhibited (Van Zelm et al. 2020), and consequently, growth and development are hampered (Acosta-Motos et al. 2017).

The perception of salt stress by the plants is followed by the induction of signaling pathways. Signaling networks are triggered that involve phytohormones, calcium (Ca^2+^), ROS, cyclic nucleotides and others. The signaling events may culminate in the modulation of expression of saltstress-responsive genes, which control the processes such as osmotic adjustment, ion transport, and ROS detoxification. Together, these strategies promote the plant’s survival under salt stress.

In signaling pathways, secondary messengers like cyclic guanosine monophosphate (cGMP) act as a link between the stimulus, and the physiological response, forming a network of molecular transducers that affect many biological processes directly or indirectly. cGMP operates as a secondary messenger in cell signaling cascades in both animals and plants. The enzymes guanylate cyclase and phosphodiesterase catalyze the synthesis and degradation of cGMP, respectively (Lucas et al. 2000). cGMP signaling plays role in diverse physiological processes in plants. To name a few, these include seed germination (Beligni and Lamattina 2000; Teng et al. 2010; Wu et al. 2013), chloroplast development (Bowler et al. 1994), hormone signaling (Jiao and Duan 2019; Penson et al. 1996), regulation of ion fluxes (Essah et al. 2003; Maathuis and Sanders 2001), regulation of biotic and abiotic stress-related gene expression (Bot et al. 2019; Durner et al. 1998; Maathuis 2006), and disease resistance (Bot et al. 2019; Hussain et al. 2016).

In animals, cGMP exerts its effects mainly through its interaction with cyclic nucleotide gated channels (CNGCs), which mediate ion fluxes across cell membranes (DeFalco et al. 2016; Demidchik et al. 2018; Leng et al. 1999) and cGMP-dependent protein kinases, which phosphorylate downstream target proteins to alter their activity (Francis et al. 2010; Shen et al. 2019). In *A. thaliana* roots, salt and osmotic stress triggers the rapid accumulation of cGMP (Donaldson et al. 2004). Moreover, exogenous application of Br-cGMP induces the expression of various monovalent cation transporter genes, and improves salt stress tolerance in Arabidopsis (Maathuis 2006; Maathuis and Sanders 2001). The latter may occur because of the effect of Br-cGMP on cation fluxes; for example, a mitigating effect of Br-cGMP on Na^+^ uptake in roots has been reported for Arabidopsis (Essah et al. 2003; Maathuis and Sanders 2001) and pepper (Rubio et al. 2003).

Tomato (*Solanum lycopersicum* L.) is one of the most popular vegetable species in the world (FAOSTAT—Crops 2020). The potential of membrane permeable Br-cGMP in protecting salt stress-exposed *S. lycopersicum* was recently explored and Br-cGMP was found to have a promoting effect on seed germination and growth parameters during salinity stress (Zhu et al. 2022). Furthermore, the use of Br-cGMP increased starch, soluble sugar, flavonoid, and lignin content, while it decreased the buildup of malondialdehyde, and boosted the activity of peroxidase (Zhu et al. 2022). However, whether the Br-cGMP-induced improvement in tomato growth was achieved (partly) through altered cation fluxes is not clear. Besides, the determination of salt stress-induced changes in cGMP content have not been studied so far in *S. lycopersicum*. The current study was conducted to determine whether salinity evokes a cGMP signal in tomato plants and to assess if the mitigating impact of Br-cGMP on tomato salt stress could be based in its ability to regulate cation fluxes.

## Materials and methods

### Plants material and growth conditions

Seeds of *S. lycopersicum* Riogrande variety were surface sterilized and grown on Murashige and Skoog (MS) medium. Two-week old seedlings were transferred to hydroponic medium containing half strength Hoagland nutrient solution (Hoagland and Arnon 1938) in 1 L plastic containers. Growth medium was renewed twice a week. Growth chamber conditions were 16/8 h day/night at 20 °C with a relative humidity of 60% and a light intensity of 120 µmol m^− 2^ s^− 1^.

### Determination of salt stress-induced cGMP content

To determine the effect of salt stress on endogenous cGMP, four-week-old plants roots were treated with 0 or 100 mM NaCl, and samples were collected at different time points (5 s, 30 s, 1 min, 5 min, 30 min, and 60 min). cGMP concentration was determined by using an immunoassay kit (RayBio cat# 68AT-cGMP-S100) according to the recommended protocol. cGMP content was determined by taking absorbance on CLARIOstar MBGLabtech spectrometer using 450 nm excitation light. The experiment was performed in three biological replicates.

### Seed germination

Seeds of *S. lycopersicum* Riogrande variety were surface sterilized and grown on three layers of filter paper in petri dishes in the growth chamber. Germination was scored under control, NaCl (50 mM, 75 mM and 100 mM), Br-cGMP (10 µM) and combinations of NaCl and Br-cGMP. Germination data were recorded daily up to five days. A seed was considered as germinated if the radicle became 1–2 mm in length. The experiment was performed in three replicates, with each replicate consisting of at least 55 seeds.

### Leaf area, fresh and dry weight determination

For determination of leaf area, fresh weight (FW) and dry weight (DW), *S. lycopersicum* plants were treated with 100 mM NaCl in the absence or presence of Br-cGMP (10 µM) for 5 days in Hoagland nutrient solution. Leaf area was determined by using graph paper, and calculated with the formula described by Afsar et al. (2020). Whole seedlings were sampled for FW and DW determination in three replicates with each replicate consisting of six seedlings. To determine the DW, the samples were oven-dried at 80 °C for 48 h. The biomass experiment was conducted with three biological replicates while that of leaf area with five biological replicates.

### Determination of Na^+^ and K^+^ influxes

Four-week-old plants were treated with 100 mM NaCl in the presence or absence of Br-cGMP (10 µM) for 5, 15, 60 and 120 min for Na^+^ and K^+^ influx determination. In each treatment four replicates were used. Ion uptake assays were carried out by using previously reported protocol (Maathuis 2006). Briefly, plants roots were washed with cold 20 mM CaCl_2_, blotted dry and fresh weight (FW) was taken. Plants material was dried in oven for > 24 h at 80 °C and reweighed to determine dry weight (DW). Next, ions were extracted from the dried plant material by adding 20 mM CaCl_2_ to the samples for 24 h. Extract was filtered, and ion concentrations were determined on a Sherwood 410 flame photometer. Ion fluxes were calculated by taking the difference between starting and end concentration divided by time.

### Determination of Na^+^ and K^+^ effluxes

Na^+^ efflux was determined in the plants grown on standard Hoagland solution supplemented with 100 mM NaCl for 3 days. The plants were then transferred to Na^+^-free solution with or without added Br-cGMP and the increase in Na^+^ ion in the efflux buffer was determined after 5 and 24 h by using flame photometry. In each treatment six replicates were used.

For K^+^ efflux the plants were grown on standard Hoagland solution. About four week old plants were transferred to K^+^-free Hoagland solution containing 0 or 10 µM Br-cGMP for 5 and 24 h. Increase in K^+^ in efflux buffer was determined by flame photometry. In each treatment eight replicates were used.

### Determination of chlorophyll content and net photosynthesis rate

Four week old plants were treated with 100 mM NaCl in the absence or presence of Br-cGMP (10 µM) for 5 days in Hoagland nutrient solution. Chlorophyll Meter (CCM-200plus; Opti-Sciences, Hudson, NH, USA) was used to measure the chlorophyll content. The fully grown fourth leaf from each plants (control and treatment) was selected for the assay. The experiment was performed in eight replicates. A portable gas exchange analyzer iFL (ADC BioScientific Ltd., Hoddesdon, UK) was used to determine the net photosynthetic rate. The experiment was performed on a day with full light intensity and sunshine (09.00 a.m.–11.00 a.m.). The net photosynthesis rate was recorded in situ using the young, completely grown leaves (third and fourth). The experiment was performed in six replicates. The assay was conducted under the following conditions: 10 cm leaf surface diameter, 352 mmol mol^− 1^ ambient atmospheric CO_2_ concentration (Cref), 1,200 mmol m^− 2^ s^− 1^ PAR, and a wide range of 4.4 to 6.6 mbar chamber water vapour pressure.

### Determination of proline content

For proline determination, 0.5 g of plant material was homogenized in10 mL of 3% aqueous sulfosalicylic acid and the homogenate was filtered through Whatman # 2 filter paper. 2 mL of filtrate was reacted with 2 mL acid ninhydrin and 2 mL of glacial acetic acid in a test tube for 1 h at 100 °C, and the reaction was terminated by placing it in an ice bath. The reaction mixture was extracted with 4 mL toluene, and mixed vigorously with a stirrer for 15–20 s. The chromophore containing toluene was aspirated from the aqueous phase, warmed to room temperature and the absorbance was recorded at 520 nm using toluene as blank. Four biological replicates were used for each treatment. The proline concentration was determined from a standard curve and calculated on a fresh weight basis as follows:

[((xg proline/mL x mL toluene) / 115.5 {xg/(xmole]/[(g sample) /5] = (xmoles proline /g of fresh weight.

### Determination of total antioxidant capacity (T-AOC)

About four-week-old *S. lycopersicum* plants were treated with 0, and 100 mM NaCl in the absence or presence of Br-cGMP (10 µM) for 5 days for the determination of total antioxidant capacity assay (T-OAC) using total antioxidant capacity assay kit (Solarbio, BC1315) according to the manufacturer’s instructions. The reagents were mixed thoroughly and reacted for 10 min, blank was set to zero with distilled water and finally the activity was determined by taking the absorbance at 593 nm on a spectrophotometer. The experiment was performed with three biological replicates.

### Electrolyte leakage assay

Electrolyte leakage (EL) from intact roots of *S. lycopersicum* was determined by using the protocol of Li et al. (2014) with some modifications. Four-week-old plants were treated with 10 µM Br-cGMP, 200 mM NaCl or a combination of both, for 2 h and EL was determined at 1 min, 5 min, 30 min, 60 min and 120 min. The roots from each treatment were collected and washed in deionized water three times to remove surface-adhered electrolytes. Initial conductivity of the deionized water (EC1) was determined by EC meter at 25 °C. Plants roots were blotted dry, weighed, and immersed in deionized water for different times and the conductivity of the bathing solution (EC2) was determined at each time point. Finally, EL was calculated by the following formula.

$${\rm{EL = }}\,\left( {{\rm{EC2 - EC1}}} \right){\rm{/FW}}$$

The experiment was conducted with five biological replicates.

### qRT-PCR

About four-week-old *S. lycopersicum* plants grown in half strength Hoagland nutrient solution were treated with 0, and 100 mM NaCl in the absence or presence of Br-cGMP (10 µM) for 24 h. Root and shoot samples were collected and immediately frozen in liquid nitrogen and stored at -80 °C. Total RNA was isolated from each tissue using RNAprep Pure Plant Plus Kit (TIANGEN, DP441) according to the manufacturer’s instructions. 1 µg RNA was reverse transcribed into cDNA using the HiScript II Q RT SuperMix for qPCR (+ gDNA wiper) (Vazyme, R223-01) and the cDNA samples were diluted 10 times for use as a template in qRT-PCR. qRT-PCR was performed using ChamQ SYBR Color qPCR Master Mix (Vazyme, Q411-02) in a 10 µL reaction. The cycling conditions comprised of a 5 min denaturation at 95 °C, followed by 40 cycles at 95 °C for 10 s, 60 °C for 30 s, and one cycle at 95 °C for 15 s, 60 °C for 60 s, and 95 °C for 15 s. Actin gene was used as internal control. The primers are listed in Table 1. The experiment was performed with three biological replicates. Relative gene expression was calculated using the 2^−ΔΔCt^ method (Livak and Schmittgen 2001).

**Table 1:** Primers used for the determination of gene expression by qRT-PCR

S. No	Gene name	Primer sequence (5 − 3)
1	*SlHAK5*	F-GTATGATGTGAC CGTGTTACG R-TCAGATCCTGTGATG CAAAGG
2	*SlNHX2*	F-CCACCGGAAGCTTGTTACAT R-CGTGCATCCTCTCCTTCTTC
3	*SlNHX3*	F-AGAAGTCTCCGGAGGAGAGG R-AGTGTTTTGGTGAGGGTTGC
4	*SlNHX4*	F-GGACACACTCAACTGCAGGA R-GGTGAGGAAGATCTGGGACA
5	*SlActin*	F-GAAATAGCATAAGATGGCAGACG R-ATACCCACCATCACACCAGTAT

### Statistical analysis

Data was statistically analyzed by paired t-test and one-way ANOVA (POSTHOC Duncan’s multiple-range) test performed using IBM SPSS statistics for Windows, V.20 (IBM Corp). The number of replicates used for each experiment are mentioned under their respective headings in the material and method section.

## Results

### NaCl treatment induces cGMP accumulation in *S. lycopersicum* roots

**Fig. 1:**
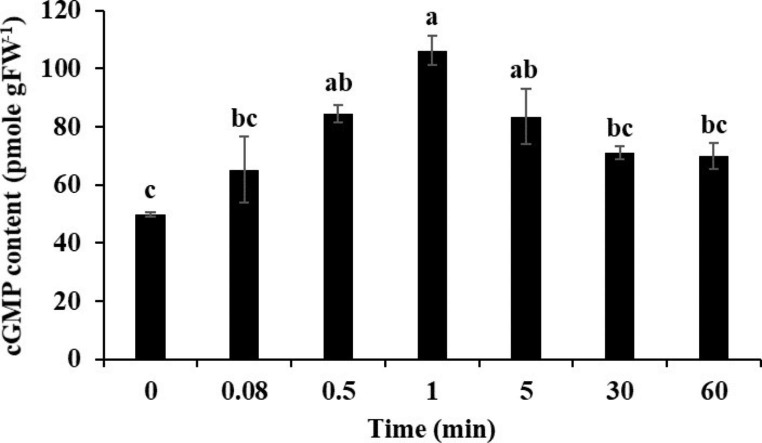
Determination of salt-stress induced cGMP content in *S. lycopersicum* roots at 0 or 100 mM NaCl at indicated time points. Values represent mean ± SE calculated from three independent biological replicates. Bars with different letters show significant differences at *P* < 0.05

NaCl stress is known to trigger cGMP accumulation in Arabidopsis (Donaldson et al. 2004). However, whether this happens in *S. lycopersicum* remains to be investigated. We therefore determined cGMP content in *S. lycopersicum* roots exposed to 100 mM NaCl at different time points (5 s to 60 min). As shown in Fig. 1, the cGMP level in *S. lycopersicum* roots rapidly increased (within seconds) after NaCl treatment compared with control. Overall, at all the tested points, the cGMP level in treated plants remained higher than the control, however, significant differences in cGMP level were recorded at 30 s, 1-minute and 5-minute time points (*P* < 0.05). The highest accumulation was detected at 1 min when cGMP level was approximately two-fold greater than the control. Together these data confirm that NaCl stress induces a rapid accumulation of cGMP in *S. lycopersicum* roots.

### Br-cGMP ameliorates the inhibitory effects of NaCl on seed germination and plant growth parameters

**Fig. 2:**
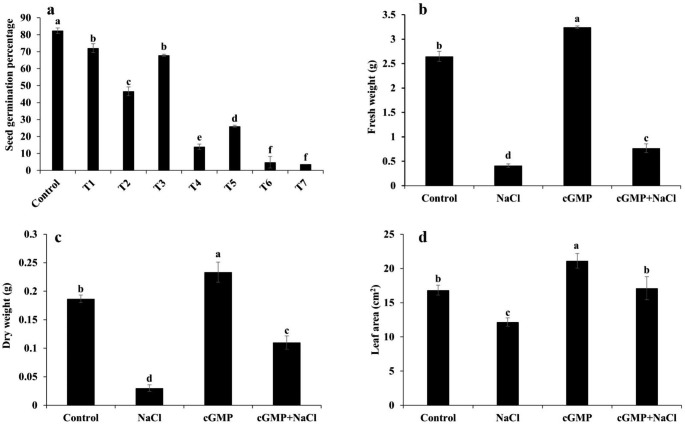
Determination of seed germination **(a)**, FW **(b)**, DW **(c)** and leaf area **(d)**. Seeds were germinated under control, T1 = Br-cGMP (10 µM), T2 = NaCl (50 mM), T3 = Br-cGMP + NaCl 50 (mM), T4 = NaCl (75 mM), T5 = Br-cGMP + NaCl (75 mM), T6 = NaCl (100 mM) and T7 = Br-cGMP + NaCl (100 mM). Data are mean ± SE of three biological replicates, with each replicate consisting of at least 55 seeds. For leaf area and biomass experiments data are mean ± SE of five and three replicates, respectively. Bars with different letters show significant differences at *P* < 0.05

The seeds of *S. lycopersicum* were germinated under control and different NaCl concentrations in the absence or presence of Br-cGMP to determine if Br-cGMP can improve seed germination during salt stress. The treatment with all three tested concentrations of NaCl (50, 75 & 100 mM) significantly reduced the seed germination in a concentration dependent manner, as compared with the control (*P* < 0.05) (Fig. 2a). The application of Br-cGMP alone showed a slight inhibitory effect on seed germination (Fig. 2a). When Br-cGMP was applied in combination with NaCl (50 mM or 75 mM), statistically significant improvement in seed germination, as compared with its respective NaCl control, was observed (*P* < 0.05) (Fig. 2a). However, at 100 mM NaCl, Br-cGMP exhibited no significant effect on germination compared with NaCl control. These data demonstrate that Br-cGMP can significantly reverse the inhibitory effects of salt stress on seed germination at NaCl concentrations up to 75 mM.

A protective effect of Br-cGMP was observed on growth parameters like leaf area, and biomass in plants under salt stress. 100 mM NaCl treatment significantly reduced fresh as well as dry biomass of the seedlings compared with the control (*P* < 0.05) (Fig. 2b, c). Addition of Br-cGMP to the medium improved fresh and dry biomass of the seedlings by around 15%. However, when Br-cGMP was supplemented to NaCl stressed seedlings, biomass increased by a much greater extent with around twofold and threefold increase in FW and DW, respectively (Fig. 2b, c). These findings show that Br-cGMP can partially reverse the inhibitory effects of NaCl stress on biomass in *S. lycopersicum* seedlings (Fig. 2b, c). A similar protective effect of Br-cGMP was also observed for plants leaf area. Compared with NaCl treatment, the addition of Br-cGMP caused about 1.5-fold increase in leaf area (Fig. 2d).

### Br-cGMP exhibits protective effects on Chlorophyll Content and Photosynthesis rate under salt stress

**Fig. 3:**
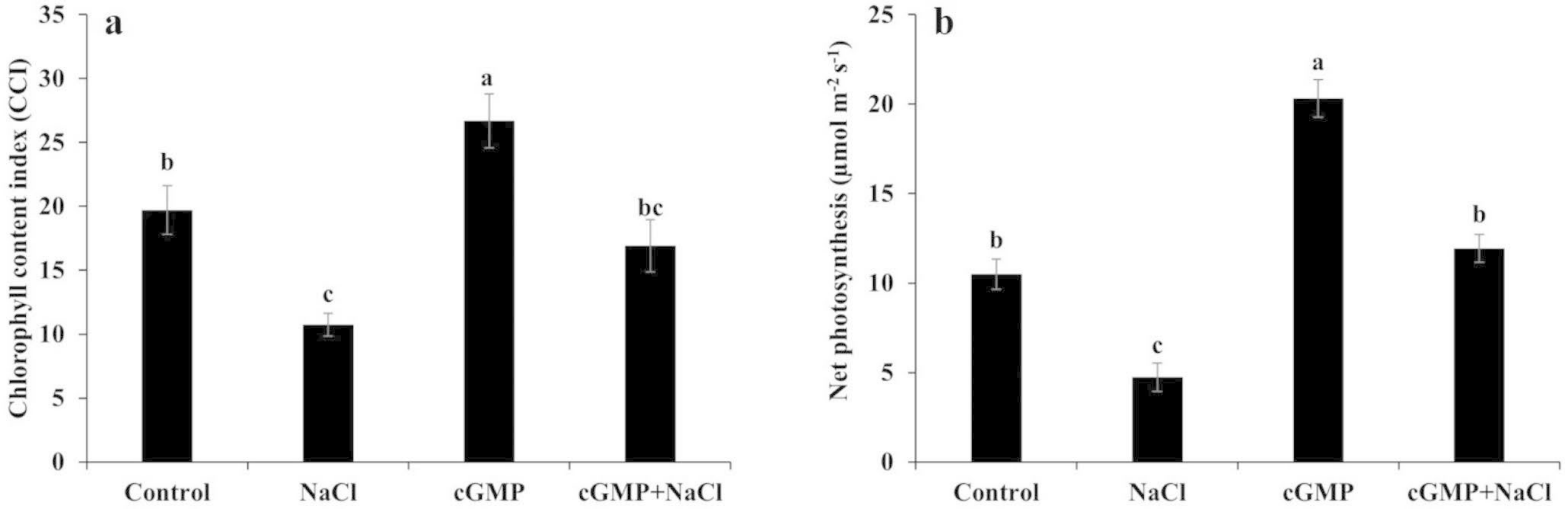
Determination of chlorophyll content **(a)** and net photosynthesis rate **(b)** in *S. lycopersicum* under different treatments. For chlorophyll content and net photosynthesis rate data are mean ± SE of eight and six biological replicates, respectively. Bars with different letters show significant differences at *P* < 0.05

Compared with the control, salt stress significantly reduced the chlorophyll content while Br-cGMP improved it (*P* ˂ 0.05) (Fig. 3a). When Br-cGMP was added along with NaCl, the former ameliorated the inhibitory effect of NaCl on chlorophyll content. The positive effect of Br-cGMP on chlorophyll prompted us to determine the net photosynthesis rate under different treatments. As expected, NaCl treatment exerted a significant inhibitory effect on net photosynthesis rate compared with the control (*P* ˂ 0.05) (Fig. 3b). Br-cGMP, on the other hand, markedly improved the net photosynthesis rate; Br-cGMP treated plants showed about two-fold higher net photosynthesis rate than control (Fig. 3b). When applied with NaCl, Br-cGMP significantly reversed the inhibitory effect of NaCl on net photosynthesis rate compared with NaCl treatment (*P* ˂ 0.05) (Fig. 3b). These data show that Br-cGMP can effectively ameliorate the inhibitory effects of salt stress on chlorophyll content and photosynthesis rate.

**Fig. 4:**
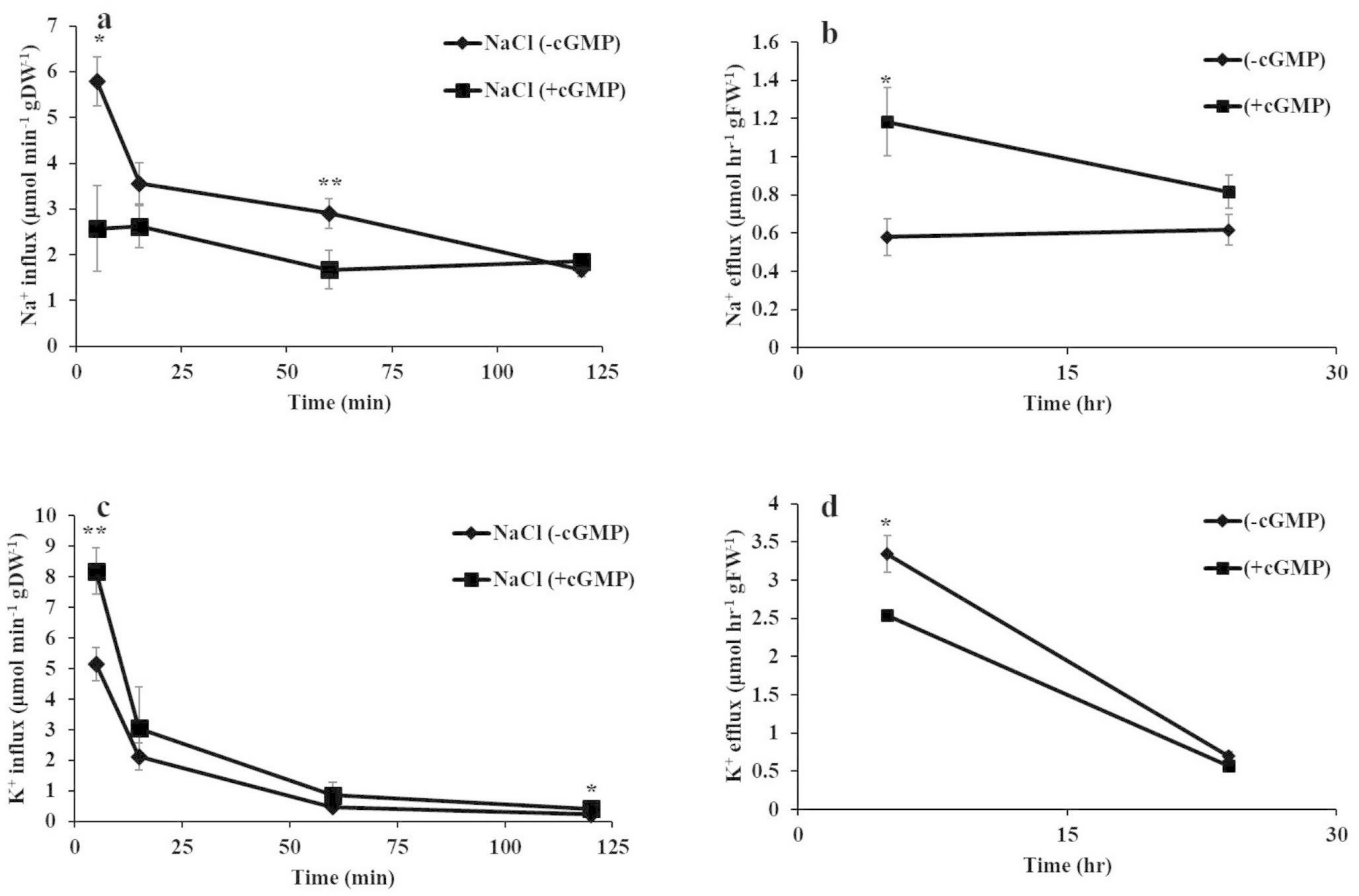
Determination of Na^+^ influx **(a)**, Na^+^ efflux **(b)**, K^+^ influx **(c)** and K^+^ efflux **(d)** in *S. lycopersicum*. Net Na^+^ influx was measured over the following time points: 5, 15, 60 and 120 min. For Na^+^ efflux, *S. lycopersicum* roots pre-loaded with 100 mM NaCl, in the absence or presence of Br-cGMP were used over the indicated time points. For Na^+^ influx and efflux data are mean ± SE of four and six biological replicates, respectively. Net K^+^ influx was measured in the absence or presence of Br-cGMP during 100 mM NaCl over the following time points: 5, 15, 60 and 120 min. K^+^ efflux determination over the indicated time points in plants. For K^+^ influx and efflux data are the mean ± SE of eight and four biological replicates, respectively. Asterisks denote significant differences at *P < 0.05 and **P < 0.01

### Br-cGMP modulates Na^+^ and K^+^ fluxes in *S. lycopersicum* under NaCl stress

Next, we were interested in investigating the underlying mechanism of the protective effect of Br-cGMP on growth parameters in NaCl treated plants. As ion channels are amongst the main downstream targets of cGMP, the ameliorative effect of Br-cGMP on plants growth parameters could possibly occur via altered (cat)ion fluxes modulated by Br-cGMP binding on these targets. We, therefore, determined the impact of Br-cGMP on influx and efflux of Na^+^ and K^+^ at different time points. In the presence of Br-cGMP, Na^+^ influx (determined at 5, 15, 60 and 120 min) was comparatively less compared with control, at all the time points except 120 min. However, the difference was significant at 5 and 60 min time points. The highest difference in Na^+^ influx was found at the 5 min time point with more than two-fold reduction compared with control (Fig. 4a). At later time points (15 and 60 min), the presence of Br-cGMP still reduced the Na^+^ influx albeit with a smaller magnitude. At the 120 min time point, the presence of Br-cGMP no longer had any significant impact on Na^+^ influx. For Na^+^ efflux, plants were pre-treated with 100 mM NaCl for 3 days and then efflux was determined in the presence or absence of Br-cGMP (Fig. 4b). The presence of Br-cGMP significantly promoted the Na^+^ efflux in *S. lycopersicum* roots at the 5 h time point with about two-fold higher Na^+^ efflux compared to roots that lacked Br-cGMP supplementation. Efflux of Na^+^ at the 24 h time point was still higher than the control but the difference was not statistically significant

**Fig. 5:**
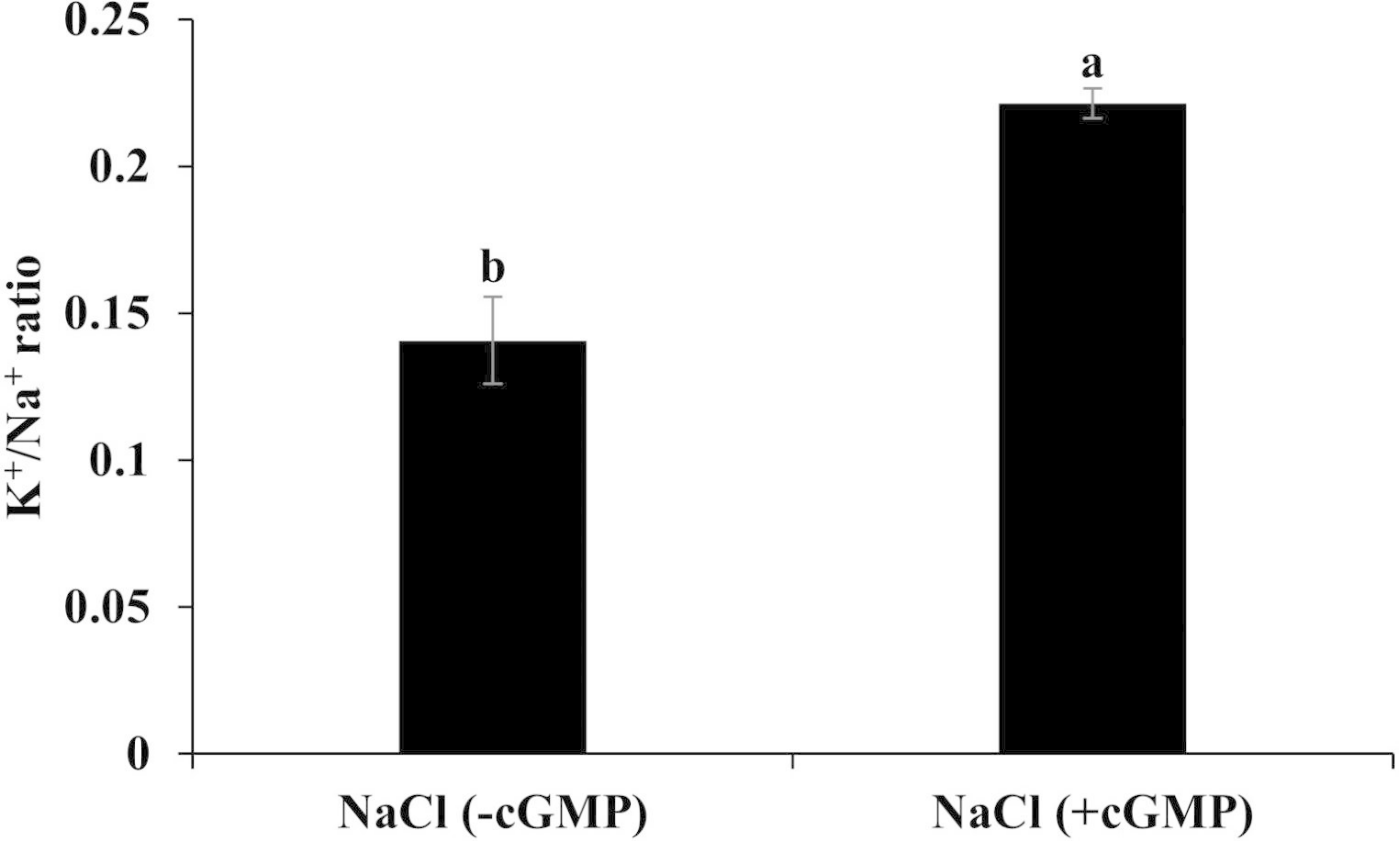
Determination of K^+^/Na^+^ ratio *S. lycopersicum* under different treatments. The presence of Br-cGMP increases the K^+^/Na^+^ ratio in roots of *S. lycopersicum* under salt stress. Data are mean ± SE of four biological replicates. Bars with different letters show significant differences at *P* < 0.05

The presence of Br-cGMP promoted K^+^ influx at all the tested time points (Fig. 4c). However, like Na^+^ influx, the difference in K^+^ influx between Br-cGMP treatment and control was more pronounced at earlier time points. The highest difference (1.6-fold) in K^+^ influx between Br-cGMP treated seedlings and control was noted at the 5 min time point. A significant reduction in K^+^ efflux was noticed in seedling roots growing in the presence of Br-cGMP at the 5 h time point compared with control (Fig. 4d) (*P* ˂ 0.05). The difference between treatment and control then reduced and at the 24 h time point, K^+^ efflux was not significantly different in the presence or absence of Br-cGMP. To sum up, these data show that Br-cGMP reduces Na^+^ influx and promotes Na^+^ efflux while it promotes K^+^ influx and reduces K^+^ efflux in NaCl treated *S. lycopersicum* roots.

### Treatment with Br-cGMP improves K^+^/Na^+^ ratio in salt stressed *S. lycopersicum*

The above data show that Br-cGMP has a significant impact on both Na^+^ and K^+^ fluxes, especially at the early stages of salinization. The net effect of the observed flux modulations is higher root tissue K^+^/Na^+^ ratio, a factor that may help stave off salt stress (Maathuis and Amtmann 1999). As shown in Fig. 5, the addition of Br-cGMP in the growth medium significantly increased the K^+^/Na^+^ ratio in salt stressed plants (*P* < 0.05) by almost doubling this parameter.

**Fig. 6:**
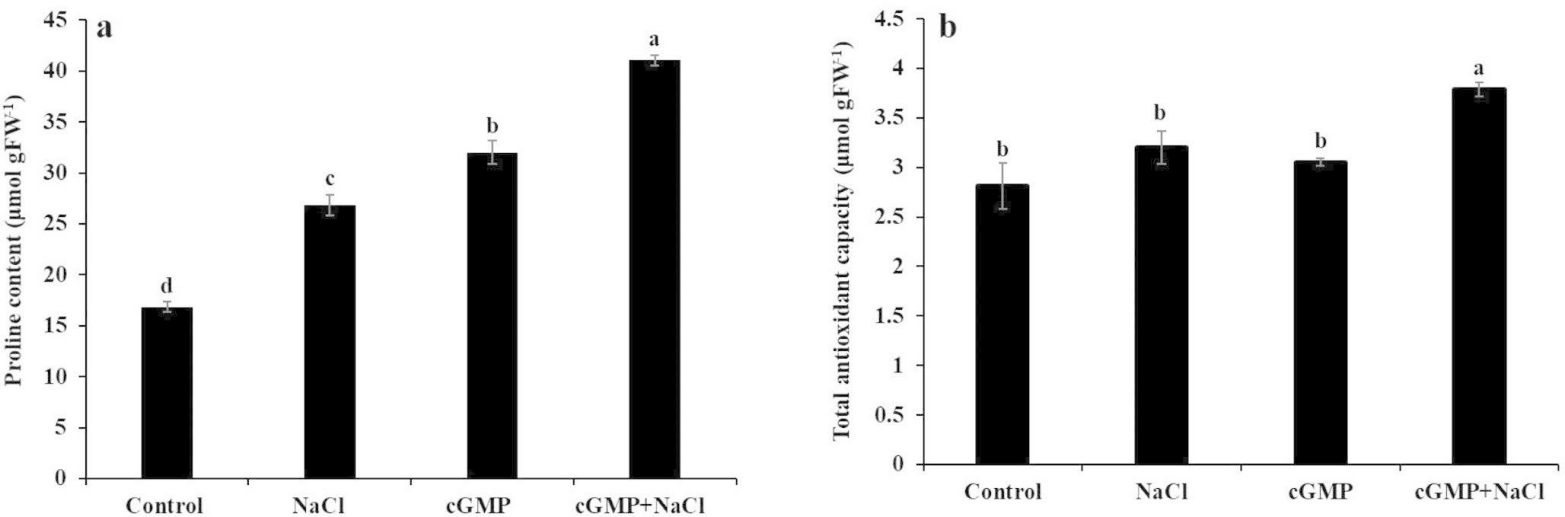
Determination of proline content **(a)** and total antioxidant capacity **(b)** under different treatments. For proline content the experiment was performed in four replicates while for total antioxidant capacity it was done with three replicates. Data are mean ± SE and bars with different letters show significant differences at *P* < 0.05

### Br-cGMP promotes Proline Accumulation and total antioxidant capacity under salt stress

Overall, significant increase in proline content was observed in NaCl- as well as Br-cGMP- treated plants, compared with control (*P* < 0.05) (Fig. 6a). When Br-cGMP and NaCl were applied as a combined treatment, the proline content was markedly higher than control and other treatments (more than two-fold increase than control).

We also determined total antioxidant capacity (T-AOC) of *S. lycopersicum* seedlings treated with 0 & 100 mM NaCl in the presence or absence of Br-cGMP. T-AOC was not significantly different in NaCl or Br-cGMP treated plants than control, however, significantly higher (*P* < 0.05) T-AOC was exhibited by plants growing under NaCl + Br-cGMP treatment compared with NaCl control (Fig. 6b). These data show that Br-cGMP improves antioxidant capacity of the plants under salt stress.

### Br-cGMP reduces NaCl stress-induced electrolyte leakage in *S. lycopersicum* roots

**Fig. 7:**
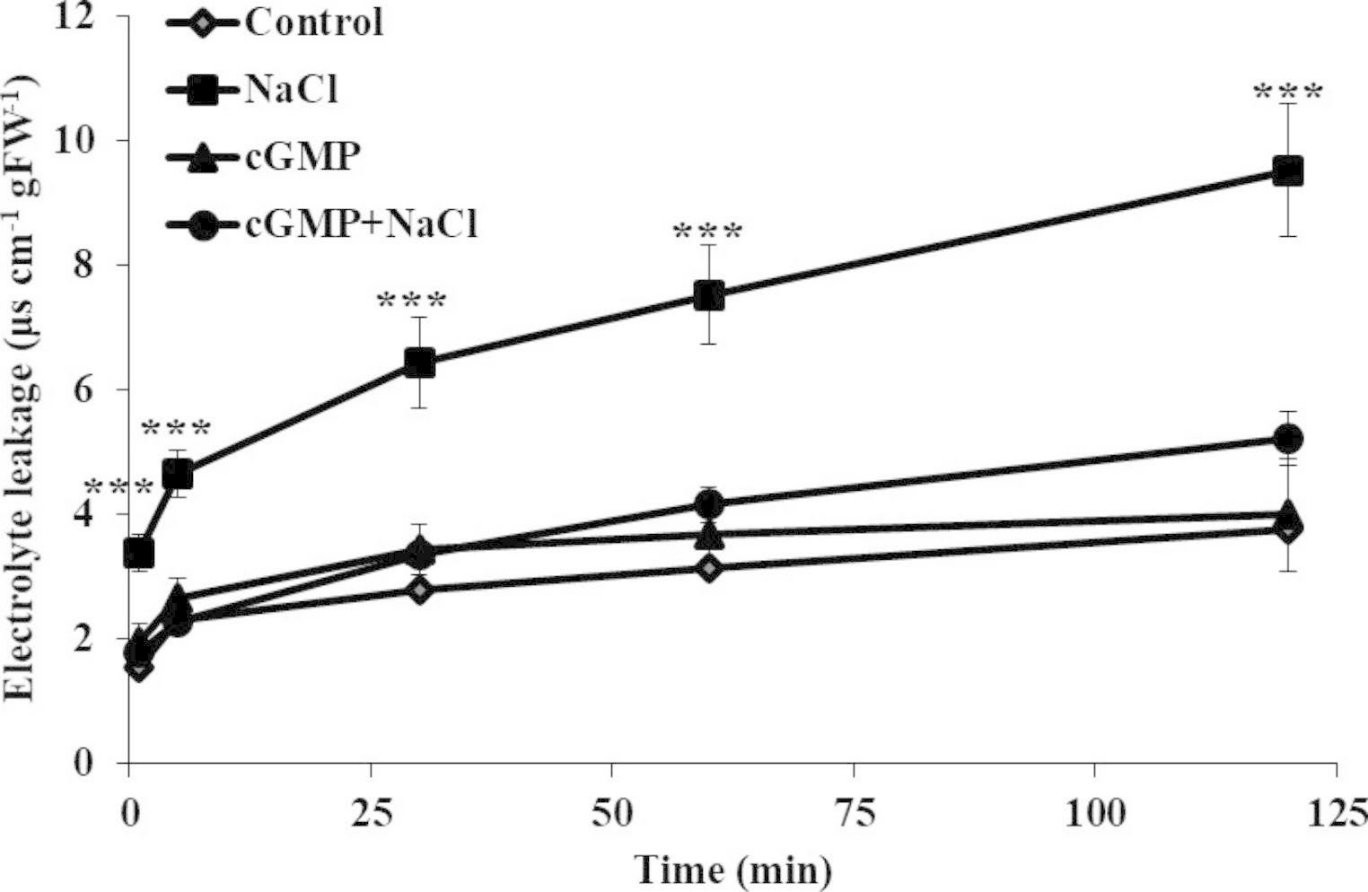
Determination of electrolyte leakage in intact *S. lycopersicum* root tissue under different treatments at 1, 5, 30, 60 and 120 min. Data are mean ± SE of five biological replicates. Data points with *** denote significant differences at *P* < 0.001

Electrolyte leakage (EL) is considered as good indicator for determination of stress-induced cell membrane damage in plants. Increased EL may cause greater influx of harmful ions while leading to loss of essential nutrients during salinity (Demidchik et al. 2014). The effect of Br-cGMP on EL in control and salinity conditions was therefore determined in the roots of intact tomato plants (Fig. 7). At all the tested time points a significant interaction was observed between NaCl and Br-cGMP treatment (*P* < 0.001). NaCl treatment caused a significant increase in EL compared with control and other treatments (*P* < 0.001). Addition of Br-cGMP to the solution significantly reduced EL caused by NaCl treatment at all the tested time points. The highest reduction in EL was observed at the 120 min time point with EL being about two-fold less than that of NaCl treatment.

### The effect of Br-cGMP on the Expression of Selected Genes involved in Na^+^ and K^+^ fluxes

**Fig. 8:**
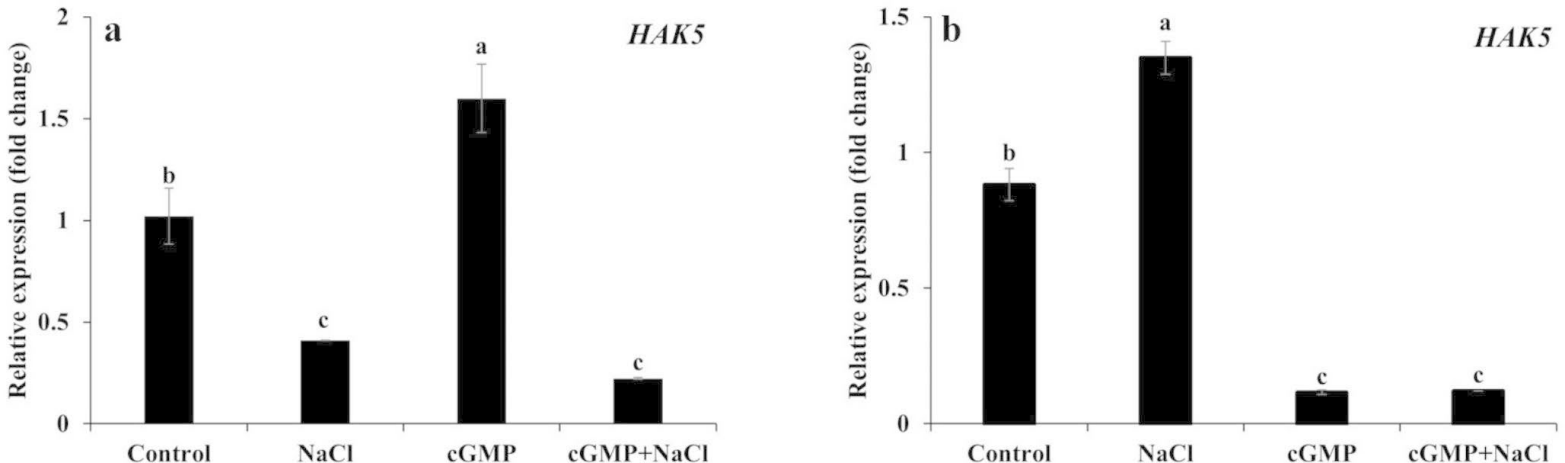
Determination of *HAK5* gene expression in *S. lycopersicum* roots **(a)** and shoots **(b)** under different treatments by using qRT-PCR. Data are mean ± SE of three biological replicates. Bars with different letters show significant differences at *P* < 0.05

NaCl and Br-cGMP exhibited a contrasting effect on *HAK5* expression in roots and shoots. Compared with control, NaCl treatment reduced the expression of the *HAK5* gene in roots but increased it in shoots (*P* < 0.05) (Fig. 8a, b). On contrary, Br-cGMP significantly induced the *HAK5* expression in roots but markedly reduced it in shoots (*P* < 0.05). However, compared to NaCl treatment, the *HAK5* expression was not different in roots under combined treatment of NaCl + Br-cGMP. In shoots, NaCl induced increase in *HAK5* expression was significantly repressed by Br-cGMP (*P* < 0.05).

**Fig. 9:**
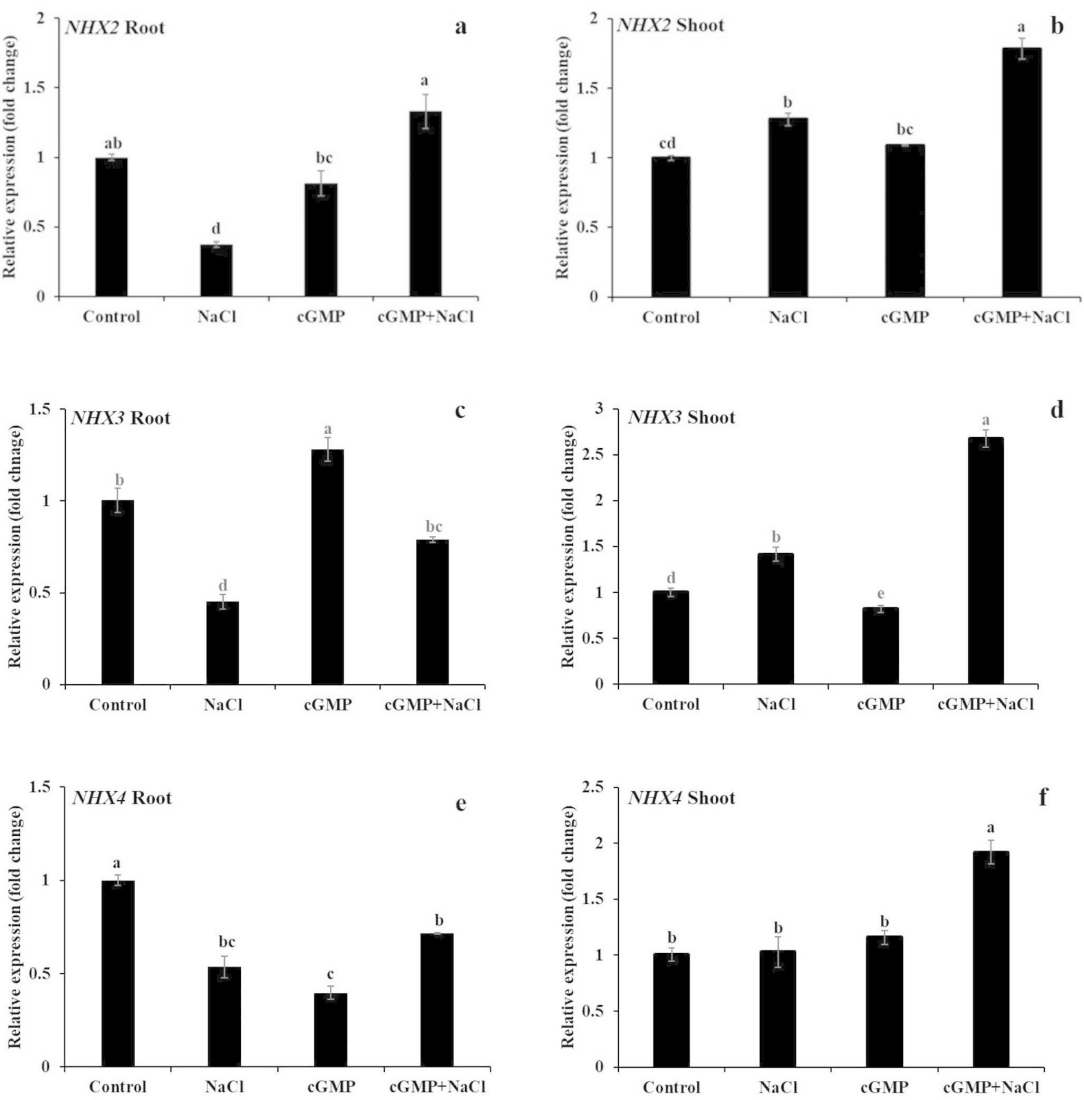
Determination of gene expression of *NHX2* gene in roots **(a)** and shoots **(b)**, *NHX3* in roots **(c)** and shoots **(d)**, *NHX4* in roots **(e)** and shoots **(f)**, under different treatments by using qRT-PCR. Data are mean ± SE of three biological replicates. Bars with different letters show significant differences at *P* < 0.05

We also determined the expression of three *NHX* isoforms in roots as well as in shoots (Fig. 9a-f). A distinct expression pattern of these genes was observed in roots and shoots. All three isoforms were significantly downregulated in roots by NaCl treatment (*P* < 0.05) while the effect of Br-cGMP on the expression of *NHXs* was variable (Fig. 9a, c, e). Importantly, the expression of *NHX2* and *NHX3* was significantly higher in roots under combined NaCl + Br-cGMP treatment compared with NaCl alone.

In shoots, NaCl treatment significantly increased the expression of *NHX2* and *NHX3* (*P* < 0.05) while Br-cGMP only had a significant effect (inhibitory) on *NHX3* expression (Fig. 9b, d, f). In shoots, under NaCl + Br-cGMP treatment, the expression of all three *NHX* isoforms was significantly higher compared with all other treatments (*P* < 0.05). These data show that supplementation with Br-cGMP increases the expression of *NHX2* and *NHX3* in salt stressed roots while it increases the expression of all three *NHX* isoforms in shoots of *S. lycopersicum* under salt stress.

## Discussion

Salt stress damages plants in a multitude of ways that include inhibition of seed germination, retarded growth, delayed development, and altered flowering and fruiting (Park et al. 2013; Quan et al. 2007). Being sessile in nature, plants must adopt different strategies to cope with salt stress. However, these adaptive measures are linked with upstream stress perception. The earliest signaling molecules generated in the plants after salt stress treatment include apoplastic Na^+^, Ca^2+^, H_2_O_2_ and cGMP indicating that these might be involved in salt stress perception and/or early stress signaling (Park et al. 2016; Shabala et al. 2015). The increase in cellular cGMP has been detected within seconds after onset of salinity and osmotic stress (Donaldson et al. 2004). In the current research we, for the first time, report a rapid salt stress-induced cGMP accumulation in the model plant *S. lycopersicum* (Fig. 1). In combination with the work by Donaldson et al. (2004), these findings suggest that a rapid, salinity-induced cGMP signal may be a common feature of the plants salt stress response.

The accumulation of cGMP is positively correlated with plants salt stress tolerance. So, the question is what the downstream mechanisms could be through which cGMP might be altering plant’s salt stress response. In the light of our findings and previously published work, cGMP seems to be involved in all three main aspects of salt stress i.e. osmotic stress, ion toxicity and oxidative stress. Below we discuss these one by one:

To mitigate water deficiency associated with osmotic stress, plants carry out osmotic adjustment, by synthesizing compatible solutes like proline and glycine betaine to maintain cell turgor and volume (Van Zelm et al. 2020). Through osmotic adjustment plants prevent dehydration stress by reducing the cytosolic osmotic potential (Jogawat 2019; Yang and Guo 2018). Under osmotic stress, proline accumulation prevents protein dehydration and denaturation in plants (Liang et al. 2013). In our study, higher proline accumulation under combined treatment with NaCl and Br-cGMP (Fig. 6a) could be one of the underlying mechanisms for cGMP-induced salt tolerance in *S. lycopersicum.*

As far as mitigation of ionic stress is concerned, cGMP seems to play diverse roles. We have demonstrated that membrane permeable Br-cGMP reduced plant’s net Na^+^ content by decreasing Na^+^ influx and increasing Na^+^ efflux while it improved net K^+^ content by increasing K^+^ influx and decreasing K^+^ efflux in salt stressed *S. lycopersicum* seedlings (Fig. 4). This was evident by a significantly improved K^+^/Na^+^ ratio in the seedlings exposed to NaCl in the presence of Br-cGMP (Fig. 5). cGMP can modulate the ion fluxes by direct binding on ion channels, and/or by regulating the transcription of ion channel encoding genes. In a previous study, Br-cGMP dependent changes in transcript level were shown to over-represent monovalent cation transporter genes including K^+^ channels, CNGCs, and monovalent antiport systems (Maathuis 2006). Several of these systems contain (putative) cyclic nucleotide binding domains and hence may be directly controlled by Br-cGMP. Regulation of CNGCs in this manner could affect Na^+^ influx since several CNGCs are known to mediate Na^+^ uptake (Gobert et al. 2006). Similarly, a plasma membrane antiporter has a cyclic nucleotide binding domain (Isayenkov et al. 2020) that could mediate a Br-cGMP-dependent activation after the onset of salinity. Our data shows that the expression of *NHX* isoforms was significantly upregulated in roots and shoots in combined NaCl and Br-cGMP treatment compared with NaCl control (Fig. 9). These data support our Na^+^ flux data (Fig. 4) and are consistent with previously published work (Maathuis and Sanders 2001). Under salt stress, excessive Na^+^ entry in plant cells leads to K^+^ loss (Park et al. 2016; Zhao et al. 2021). The HAK/KT/KUP transporters like *HAK5* play important role in maintaining optimum Na^+^/K^+^ during salt stress. *HAK5* is mainly involved in high affinity K^+^ uptake in plants roots (Li et al. 2018). In addition to its role in high-affinity K^+^ uptake, rice *HAK5* is also involved in the root-to-shoot K^+^ transport and salinity tolerance (Yang et al. 2014). We have reported that Br-cGMP had a pronounced effect on *HAK5* expression in *S. lycopersicum* shoots; Br-cGMP alone or in combination with NaCl significantly downregulated the expression of the *HAK5* gene (Fig. 8). This could be due the fact that salt stressed plants growing in the presence of Br-cGMP had higher K^+^ (as manifested by increased K^+^ / Na^+^ ratio) so this could have led to the downregulation of the *HAK5* gene. The expression of *HAK5* was downregulated by salt stress in glycophytes like Arabidopsis and tomato (Nieves-Cordones et al. 2007, 2008, 2010). This reduction was found to be closely related to salinity-induced membrane depolarization which plays crucial role in regulating *HAK5* expression in tomato (Nieves-Cordones et al. 2008). The downregulation of *HAK5* expression in shoots by Br-cGMP could also be due to Br-cGMP-induced modulation of cation fluxes, consequently affecting the membrane potential. It is likely that Br-cGMP-induced K^+^ fluxes might be taking place through transport protein(s) other than *HAK5*. Further experiments would be needed to clearly understand the molecular mechanisms involved in cGMP-induced changes in plant’s K^+^ content under salt stress.

Besides direct roles in modulating ion fluxes, cGMP has been reported to play an indirect role through interaction with other signaling intermediates. It has been reported that cGMP, through hydrogen peroxide (H_2_O_2_) and Ca^2+^, modulates the salt resistant pathway in Arabidopsis roots (Li et al. 2011). cGMP was able to reduce salt stress induced injury by increasing the activity of plasma membrane (PM) ATPase; latter helping to maintain ion homeostasis (Li et al. 2011). The cGMP pathway has been shown to interact with phytohormone ethylene in mediating salt stress resistance. Li et al. (2014) has presented a signaling network involving ethylene and cGMP in the salt resistance pathway of Arabidopsis roots. cGMP, via inducing ethylene production, alleviated NaCl-induced injury by maintaining a lower Na^+^/K^+^ ratio and increasing PM H-ATPase activity which ultimately resulted in maintaining ion homeostasis.

Salt stress triggers ROS production which damages the membranes, subsequently increasing the electrolyte leakage (Demidchik et al. 2003, 2010, 2014). The ROS detoxification in plants is carried out through enzymatic and non-enzymatic antioxidants (Foyer and Noctor 2005; Mittler 2002; Mittler et al. 2004). In the current study the supplementation of Br-cGMP to salt stressed plants induced a significantly higher proline and total antioxidant capacity as compared with NaCl control (Fig. 6). It has been recently reported that cGMP enhances POD activity in salt treated seedlings of cherry tomato (Zhu et al. 2022). It seems that cGMP improves plant’s ROS detoxification capability through enzymatic as well as non-enzymatic antioxidants. Plants equipped with better ROS scavenging are expected to undergo less membrane damage. In fact, consistent with this notion, addition of Br-cGMP significantly lowered NaCl-induced electrolyte leakage in *S. lycopersicum* (Fig. 7), showing less membrane damage.

The ameliorative effect of Br-cGMP on seed germination, growth parameters, pigments and photosynthesis in salt stressed plants could be attributed to plant’s ability to undergo osmotic adjustment, carry out toxic ion detoxification and through effective ROS scavenging ability. The overall effect of these mechanisms is reflected in enhanced salt stress tolerance by Br-cGMP augmented plants. Besides, a few of these parameters could be modified directly by Br-cGMP through other mechanisms. For example, seed germination is inhibited by salt stress due to ABA accumulation (Wang et al. 2015) while cGMP reduces the sensitivity of seed germination to ABA, thus promoting seed germination under salt stress (Teng et al. 2010). Regarding photosynthesis, cGMP can affect it in different ways. First, cyclic GMP, along with calcium, stimulates the synthesis of fully developed chloroplasts, and stimulates the production of photoprotective anthocyanins (Bowler et al. 1994). Moreover, several enzymes of the Calvin cycle were amongst the cellular proteins interacting with cyclic nucleotides (Donaldson et al. 2016), pointing to the possible involvement of cGMP in this process. Another way by which cGMP is involved in photosynthesis is through its regulatory effects on stomatal movements (Cousson 2003; Desikan et al. 2004; Dubovskaya et al. 2011; Honda et al. 2015; Hossain et al. 2014; Joudoi et al. 2013; Neill et al. 2003; Pharmawati 1999; Pharmawati et al. 2001).
