# Correction to: Cyclic guanosine monophosphate improves salt tolerance in *Solanum lycopersicum*

**DOI:** 10.1007/s10265-023-01497-x

**Published:** 2023-10-03

**Authors:** Gulnaz Bibi, Iqra Shafique, Sartaj Ali, Raza Ahmad, Mohammad Maroof Shah, Tatheer Alam Naqvi, Iftikhar Zeb, Frans J. M. Maathuis, Jamshaid Hussain

**Affiliations:** 1 Department of Biotechnology, COMSATS University Islamabad, Abbottabad Campus, University Road, Tobe Camp, Abbottabad, 22060 Pakistan; 2 Biology Department, University of York, York, YO10 5DD UK

**Correction to: Journal of Plant Research**

10.1007/s10265-023-01487-z

In the original publication of the article, Fig. 6 was published under the section “Br-cGMP modulates Na + and K + fluxes in S. lycopersicum under NaCl stress”. In the updated version, it has been placed correctly under the section “Br-cGMP promotes Proline Accumulation and total antioxidant capacity under salt stress”.

In addition, legends of Figs. 4, 5 and 6 were published incorrectly and the correct figure legends are provided below.

**Fig. 4**: Determination of Na^+^ influx (a), Na^+^ efflux (b), K^+^ influx (c) and K^+^ efflux (d) in *S. lycopersicum*. Net Na^+^ influx was measured over the following time points: 5, 15, 60 and 120 min. For Na^+^ efflux, *S. lycopersicum* roots pre-loaded with 100 mM NaCl, in the absence or presence of Br-cGMP were used over the indicated time points. For Na^+^ influx and efflux data are mean ± SE of four and six biological replicates, respectively. Net K^+^ influx was measured in the absence or presence of Br-cGMP during 100 mM NaCl over the following time points: 5, 15, 60 and 120 min. K^+^ efflux determination over the indicated time points in plants. For K^+^ influx and efflux data are the mean ± SE of eight and four biological replicates, respectively. Asterisks denote significant differences at *P < 0.05 and **P < 0.01.

**Fig. 5**: Determination of K^+^/Na^+^ ratio *S. lycopersicum* under different treatments. The presence of Br-cGMP increases the K^+^/Na^+^ ratio in roots of *S. lycopersicum* under salt stress. Data are mean ± SE of four biological replicates. Bars with different letters show significant differences at *P* < 0.05.

**Fig. 6**: Determination of proline content (a) and total antioxidant capacity (b) under different treatments. For proline content the experiment was performed in four replicates while for total antioxidant capacity it was done with three replicates. Data are mean ± SE and bars with different letters show significant differences at *P* < 0.05.

Further, under the section, “Br-cGMP modulates Na + and K + fluxes in S. *lycopersicum* under NaCl stress”, figure numbering in the text was published incorrectly as Fig. 5a, b, c and d. It has been corrected to Fig. 4a, b, c and d in the corrected version.

Similarly, under the section, “Treatment with Br-cGMP improves K+/Na + ratio in salt stressed *S. lycopersicum*”, figure numbering in text was published incorrectly as Fig. 6. It has been corrected to Fig. 5.

Likewise, under the section, “Br-cGMP promotes Proline Accumulation and total antioxidant capacity under salt stress”, figure numbering in the text was published incorrectly as Fig. 4a and b. It has been corrected to Fig. 6a and b.

Further, under the Discussion section, on 3rd paragraph, figure numbering in text was published incorrectly as Fig. 4a. The correct numbering should read as Fig. 6a and the correct sentence should read as “In our study, higher proline accumulation under combined treatment with NaCl and BrcGMP (Fig. 6a) could be one of the underlying mechanisms for cGMP-induced salt tolerance in *S. lycopersicum*.”

Under the same section, on 4th paragraph, figure numbering should be corrected to Figs. 4 and 5. Hence, the correct sentence should read as follows, “As far as mitigation of ionic stress is concerned, cGMP seems to play diverse roles. We have demonstrated that membrane permeable Br-cGMP reduced plant’s net Na + content by decreasing Na + influx and increasing Na + efflux while it improved net K + content by increasing K + influx and decreasing K + efflux in salt stressed *S. lycopersicum* seedlings (Fig. 4). This was evident by a significantly improved K+/Na + ratio in the seedlings exposed to NaCl in the presence of Br-cGMP (Fig. 5)”.

In addition, under the same section, Fig. 5 should be corrected to Fig. 4 in the following sentence, “These data support our Na + flux data (Fig. 4) and are consistent with previously published work (Maathuis and Sanders 2001).”

Finally, under the same section, Fig. 4 should be corrected to Fig. 6 in the following sentence “In the current study the supplementation of Br-cGMP to salt stressed plants induced a significantly higher proline and total antioxidant capacity as compared with NaCl control (Fig. 6).”

The original article has been updated.

